# Differential transcriptomic analyses revealed genes and signaling pathways involved in iono-osmoregulation and cellular remodeling in the gills of euryhaline Mozambique tilapia, *Oreochromis mossambicus*

**DOI:** 10.1186/1471-2164-15-921

**Published:** 2014-10-23

**Authors:** Siew Hong Lam, Eei Yin Lui, Zhengjun Li, Shaojiang Cai, Wing-Kin Sung, Sinnakaruppan Mathavan, Toong Jin Lam, Yuen Kwong Ip

**Affiliations:** NUS Environmental Research Institute, National University of Singapore, 5A Engineering Drive, 117411 Singapore, Singapore; Department of Biological Science, National University of Singapore, S3-Level 5, 14 Science Drive 4, 117543 Singapore, Singapore; School of Computing, National University of Singapore, 13 Computing Drive, 117417 Singapore, Singapore; Genome Institute of Singapore, 60 Biopolis Street, 138672 Singapore, Singapore

**Keywords:** Transcriptomic analysis, RNA sequencing, Gene expression, Signaling pathways, Iono-osmoregulation, Cellular remodeling, Euryhaline fish gills, Mozambique tilapia *Oreochromis mossambicus*

## Abstract

**Background:**

The Mozambique tilapia *Oreochromis mossambicus* has the ability to adapt to a broad range of environmental salinities and has long been used for investigating iono-osmoregulation. However, to date most studies have focused mainly on several key molecules or parameters hence yielding a limited perspective of the versatile iono-osmoregulation in the euryhaline fish. This study aimed to capture transcriptome-wide differences between the freshwater- and seawater-acclimated gills of the Mozambique tilapia.

**Results:**

We have identified over 5000 annotated gene transcripts with high homology (E-value <1.0E-50) to human genes that were differentially expressed in freshwater- and seawater-acclimated gills of the Mozambique tilapia. These putative human homologs were found to be significantly associated with over 50 canonical signaling pathways that are operating in at least 23 biological processes in relation to branchial iono-osmoregulation and cellular remodeling. The analysis revealed multiple signaling pathways in freshwater-acclimated gills acting in concert to maintain cellular homeostasis under hypo-osmotic environment while seawater-acclimated gills abounded with molecular signals to cope with the higher cellular turn-over rate, energetics and iono-regulatory demands under hyper-osmostic stress. Additionally, over 100 transcripts encoding putative inorganic ion transporters/channels were identified, of which several are well established in gill iono-regulation while the remainder are lesser known. We have also validated the expression profiles of 47 representative genes in freshwater- and seawater-acclimated gills, as well as in hypersaline-acclimated (two-fold salinity of seawater) gills. The findings confirmed that many of these responsive genes retained their expression profiles in hypersaline-acclimated gills as in seawater-acclimated gills, although several genes had changed significantly in their expression level/direction in hypersaline-acclimated gills.

**Conclusions:**

This is the first study that has provided an unprecedented transcriptomic-wide perspective of gill iono-osmoregulation since such studies were initiated more than 80 years ago. It has expanded our molecular perspective from a relatively few well-studied molecules to a plethora of gene transcripts and a myriad of canonical signaling pathways driving various biological processes that are operating in gills under hypo-osmotic and hyper-osmotic stresses. These findings would provide insights and resources to fuel future studies on gill iono-osmoregulation and cellular remodeling in response to salinity challenge and acclimation.

**Electronic supplementary material:**

The online version of this article (doi:10.1186/1471-2164-15-921) contains supplementary material, which is available to authorized users.

## Background

The ability of the euryhaline fish to adapt to a broad range of aquatic environmental salinities is an interesting phenomenon of adaptive homeostatic response that is crucial for survival and therefore provides an excellent model for investigating iono-osmoregulation and its molecular plasticity. Understanding the euryhalinity of fish could provide evolutionary perspective into the adaptation of fish in various aquatic environments including the great divide between the freshwater and marine species [1] while probing deeper into its molecular plasticity would yield insight into the versatile iono-osmoregulatory mechanism and control [2, 3]. In turn, it would enhance our understanding of iono-osmoregulation from the molecular, cellular, tissue-organ, systems to whole-organism levels, which may contribute to fishery, environmental and even biomedical research. To be euryhaline, the fish must be able to cope with salt depletion and water gain when it is in freshwater (FW), and deal with osmotic water loss and salt gain when it is in seawater (SW) or hypersaline water (HSW). Euryhalinity therefore requires extreme versatility in the switching of iono-osmoregulatory mechanisms and cellular remodeling in iono-osmoregulatory tissue-organ in response to changes in environmental salinity [4].

The fish gill is a major iono-osmoregulatory organ and is therefore a principal site for entry and departure of ion and water molecules between its internal fluid and its external environment [5]. As part of the iono-osmoregulatory mechanism, the gills are actively sequestering salt and minimizing water gain when in FW environment, while in SW the gills are actively extruding excess salt and minimizing water loss into the external environment. The ability to sequester or extrude salt against concentration gradient in FW and SW, respectively, are performed by specialized distinct cell types which occupy the epithelial layer of the gill [5, 6]. Embedded in the plasma membrane of these cells are specific transporters and channels that worked collaboratively to ensure selective movement of ions across the epithelial layer of the gill [4–8]. For a euryhaline fish to move from FW to SW environment or vice versa, it would have to remodel the cellular landscape of the gill by changing cell types along with their transporters/channels and intercellular organization in order to cope with the reversed iono-osmoregulatory challenges. It is this versatility in cellular remodeling along with the transporters and channels within the gills in response to salinity challenge that is partly responsible for euryhalinity in fish.

The Mozambique tilapia *Oreochromis mossambicus* has versatile iono-osmoregulatory ability that enables it to live in both FW (salinity at 0 ppt) and SW (salinity at 30–35 ppt), and can tolerate extreme hypersalinity of up to 80–120 ppt, i.e. about 3–4 fold the salinity of SW. This makes Mozambique tilapia among the few remarkable successful teleost species inhabiting extreme hypersaline environment [9, 10]. The versatility of the tilapia to iono-osmoregulate across a broad range of extreme salinity has been the focus of many studies ranging from physiological, morphological, biochemical to molecular levels. Recent reviews had documented studies investigating iono-osmoregulatory changes in, although not limited to, tilapia gills and interested readers are advised to refer to them for details [7, 8, 11]. While many of the studies had been elegantly executed, most of them had focused mainly on several key molecules or parameters hence yielding important, albeit limited, perspectives of the iono-osmoregulation changes in response to different environmental salinities. An earlier attempt to capture a broader molecular perspective employing suppression subtractive hybridization approach reported 20 genes associated with 6 molecular processes with immediate response to salinity challenge in *O. mossambicus* gills [12]. The study was focused on immediate response to salinity challenge that occurred few hours after transfer to SW hence detecting only early gene responses and not those involved in gill salt transport in acclimated state. Moreover, the suppression subtractive hybridization approach is low-throughput and is only sensitive to detect high abundant transcript changes, and the challenges of annotating partial sequences in non-model organism at the time of the study may have limited the identification of more genes. This was similarly reflected in two other osmoregulation studies on non-model euryhaline fish species subjected to salinity challenge; one study on the European eel *Anguilla anguilla,* employed a combination of subtracted libraries and cDNA arrays, identified 95 known genes differentially expressed in various tissues of eel acclimated to SW [13], while another early response study on estuarine goby *Gillichthys mirabilis* employed cDNA arrays and identified 168 known genes that were differentially expressed within the first 12 hours of salinity challenge [14]. A recent study [15] reported the Japanese eel *Anguilla japonica*’s gill transcriptome generated from a single library consisting of samples pooled together from FW and after a short-term 6 hour exposure in SW, hence did not provide transcriptomic differences between gills acclimated in FW and SW; the study focused mainly on the proteome differences of short term salinity challenge. Therefore, to date there has not been a study that successfully captures the euryhaline teleost gill transcriptomic-wide changes in response to salinity.

In this study, we employed next-generation sequencing (NGS) technology to sequence and *de novo* assembled the gill transcriptome of euryhaline Mozambique tilapia acclimated in FW and SW environments. The aim of this study is to identify transcriptomic differences associated with changes in FW- and SW-acclimated gills within a single species. With increasing quality throughput in NGS technology and improvement in algorithm used for *de novo* sequence assembly, it is possible to capture the gill transcriptome in almost its entirety. Our differential transcriptomic analyses revealed a plethora of transcripts including many that are encoding inorganic ion transporters/channels and a myriad of signaling pathways involved in cellular remodeling that are operating in FW and SW acclimated tilapia gill. Since Mozambique tilapia has the ability to tolerate hypersalinity, we performed additional experiments using a new batch of fish acclimated to FW (0 ppt), SW (30 ppt) and HSW (70 ppt) and validated the NGS data and obtained further insights on how representative genes behave in gills subjected to extreme hypersalinity challenge. Although changes in transcript abundance need not translate to corresponding changes in protein abundance, nevertheless, this study has provided an unprecedented view into the transcriptomic changes and identified many transcripts encoding proteins that could potentially alter biological signaling and processes in gills under hypo- and hyper-osmostic environments. To our best knowledge, this is the first NGS study that had provided a differential transcriptome-wide perspective in gills of a euryhaline fish acclimated to FW and SW.

## Results and discussion

### Sequencing and *de novo*assembly of FW and SW gill transcriptomes

**Figure 1:**
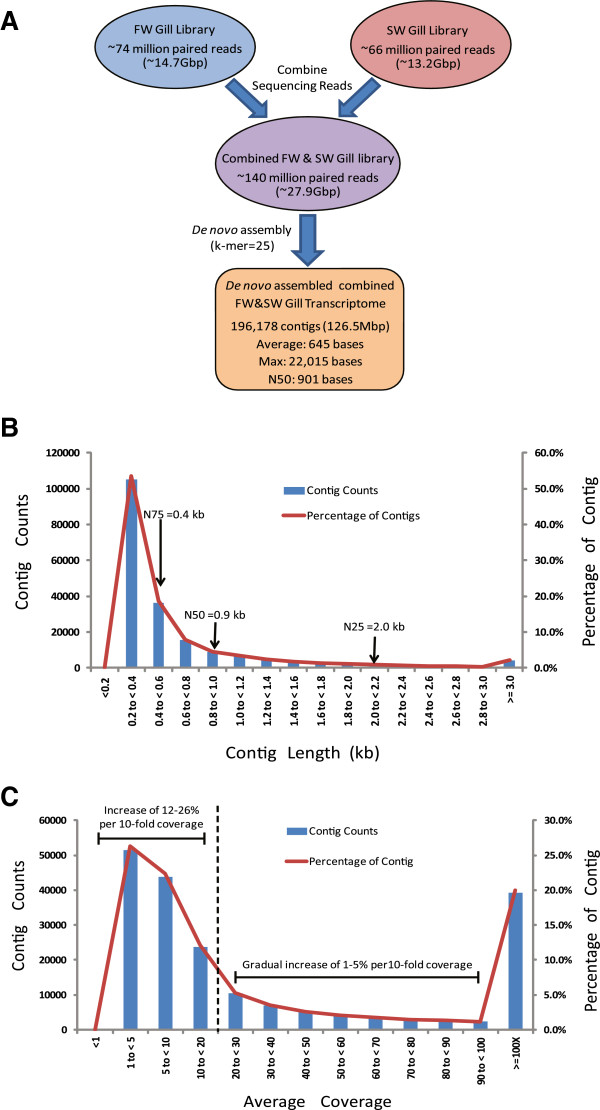
**Summary statistics of sequencing output and** ***de novo*** **assembly of the combined FW&SW gill transcriptome of** ***Oreochromis mossambicus*** **. (A)** Schematic overview of sequencing outputs and *de novo* assembly of the combined FW&SW gill transcriptome using 2X101-bp paired-end reads from freshwater (FW) and seawater (SW) gill libraries. **(B)** Distribution (number and percentage) of assembled contigs with different contig length (kb). Included are contig lengths for N-25, N-50 and N-75. **(C)** Distribution (number and percentage) of assembled contigs with different average coverage. Note steep and gradual change in contig counts or proportion with average coverage less than and above 20-fold (delineated by dotted line).

To identify transcriptomic differences associated with changes in FW- and SW-acclimated gills, we sampled gill tissues from Mozambique tilapias that had acclimated in their respective FW and SW environments for 3 weeks. We extracted total RNA from the gill tissues of the FW and SW groups and pooled four of the highest quality RNA samples from each group for preparation of the respective FW and SW cDNA libraries. Each cDNA library was prepared from fragmented poly-A RNAs that were enriched from the pooled total RNAs for paired-end sequencing (2X101bp) on Illumina Hiseq 2000 platform (see Methods). After sequencing and quality checks, more than 74 million paired reads and 66 million paired reads were obtained from the FW gill and SW gill cDNA libraries, respectively (Figure 1A). Since there is no genome assembly for *O mossambicus*, we assembled a common reference gill trancriptome using the total 140 million paired reads combined from both FW gill and SW gill libraries to serve as a reference for subsequent annotation and read mapping of transcript expression.

The combined FW&SW gill transcriptome was generated by performing *de novo* assembly using CLC Bio Genomics Workbench (version 5.1) on the combined FW&SW library [see Methods]. A total of 196,178 contigs were assembled which corresponded to a total contig length of approximately 126.5Mbp (Figure 1A). The average contig length was 645 bp and the N50 contig length was 901 bp. The contig length distribution ranged from 120 to 22,015 bp. With the exception of 275 contigs, all assembled contigs were longer than 200 bp in length (Figure 1B). Approximately 20.0% of the contigs have an average coverage of 100-fold or greater, and a cumulative 39.3% have 20-fold or greater, while the remaining have less than 20-fold average coverage (Figure 1C). It was observed that the contig count increased gradually (1-5% for every 10-fold coverage increment) between average coverage 20 to 100-fold but had increased markedly (12% and 26% for every 10-fold coverage increment) when average coverage is below 20-fold (Figure 1C). This indicates that a large proportion of the contigs was assembled from relatively low number of reads as the software attempted to maximise contig assembly. Since low coverage contigs are more susceptible to noise and spurious assembly, it could have contributed to the sudden large increase in contig count observed when average coverage is below 20-fold. Therefore, 20-fold average coverage appeared to be a good cut-off for greater confidence in the quality of the assembled contigs.

### *In silico*evaluation of combined FW&SW gill transcriptome

**Figure 2:**
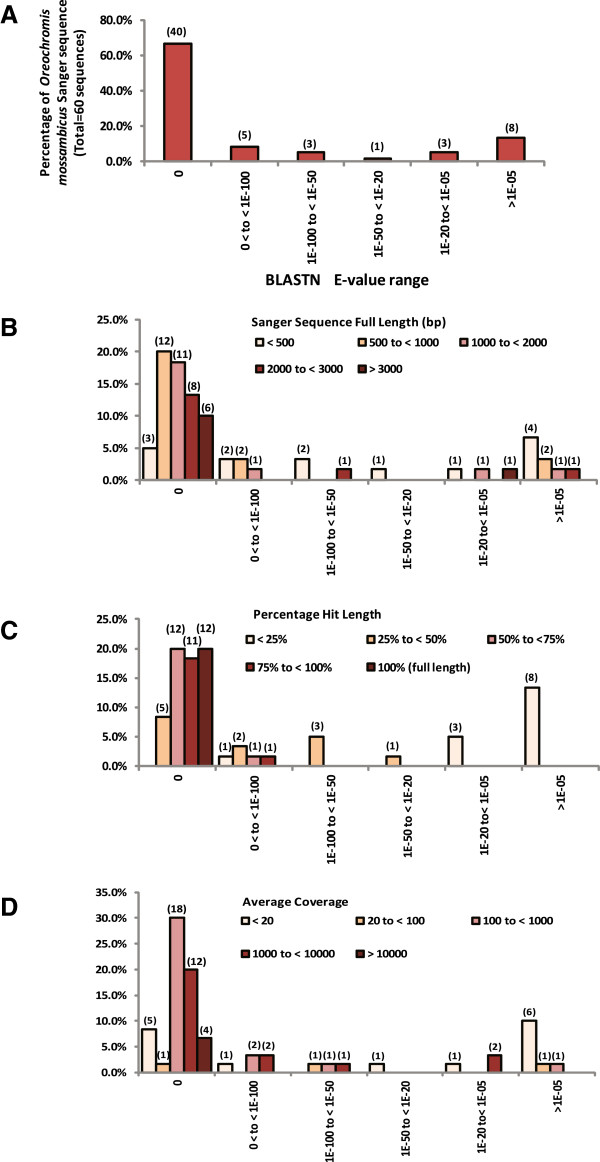
***In silico*** **evaluation of the combined FW&SW gill transcriptome using BLASTN with 60 full-length Sanger coding sequences of** ***Oreochromis mossambicus*** **. (A)** Percentage distribution (number) of the 60 Sanger sequences with respective best hit grouped within different BLASTN E-value range. The x- and y-axes labels in panel **(A)** applied to all other panels. **(B)** Percentage distribution (number) of the 60 Sanger sequences with different percentage hit length grouped within different BLASTN E-value range. **(C)** Percentage distribution (number) of the 60 Sanger sequence with different full length sizes (bp) grouped within different BLASTN E-value range. **(D)** Percentage distribution (number) of the 60 Sanger sequences with respective best hit having different average coverage grouped within different BLASTN E-value range.

To further evaluate the quality of the assembled contigs, we downloaded 60 Sanger-sequenced complete coding sequences of *O. mossambicus* from NCBI database and we performed BLASTN using these sequences against the combined FW&SW gill transcriptome (see Methods). The findings are summarized in Figure 2 (details in Additional file 1). We first described the distribution of the 60 Sanger full-length sequences based on the BLASTN E-value of the best hit (Figure 2A). Next, within different BLASTN E-value groups, the distribution of the Sanger sequences were further partitioned into groups based on their full-length size (Figure 2B), the percentage hit length (Figure 2C) and average coverage of the best hit (Figure 2D). These distributions would provide an overview on the representation of various full-length coding sequences within the combined FW&SW gill transcriptome.

Among the 60 Sanger sequences, 40 (67%) had excellent hits (E-value = 0) aligned with the assembled contigs while 8 (13%) had good hits (0 < E-value < 1.0E-50), and the remaining 12 sequences (20%) had lower homology hits (E-value > 1.0E-25) (Figure 2A; Additional file 1). The findings indicate that 80% (48/60) of the Sanger sequences were represented within E-value < 1.0E-50. Among the 40 Sanger sequences with excellent hits (E-value = 0), these full-length sequences are of various sizes (423 to 3456 bp) which 25 out of 40 are longer than 1000 bp compared to 2 out of 8 for Sanger sequences with good hits (0 < E-value < 1.0E-50) (Figure 2B). Taken together, more than half (27/48) of the Sanger sequences with hits within E-value < 1.0E-50 were greater than 1000 bp which further suggests that both short and long coding sequences were represented within the assembly. Moreover, it was found that among the 40 Sanger sequences with excellent hits (E-value = 0), 12 of the coding sequences had their full length in perfect (100%) alignment with the best hit (assembled contigs) while 11 had greater than 75% of their coding sequence aligned and the remaining 17 coding sequences had less than 75% aligned (Figure 2C; Additional file 1). Although these assembled contigs had less than 100% alignment because they were shorter than the full-length coding sequences, the average hit length of the aligned regions was about 1082 bp (range 360–2558 bp; Additional file 1). The analysis indicates that full-length and long partial length transcripts of *O. mossambicus* were represented in the assembled contigs. It was also noted that 34 out of the 40 Sanger sequences had excellent hits (E-value = 0) with average coverage >100-fold, compared to 6 out of 8 for Sanger sequences that had good hits (0 < E-value < 1.0E-50) (Figure 2D). This suggests that many of the *O. mossambicus* sequences represented within 1.0E-50 have high average coverage. Full-length representation by the assembled contigs included Sanger sequences of 459 bp to 3456 bp and had average coverage of 250 to 29,000-fold (Additional file 1).

Those Sanger sequences that had lower homology hits (E-value > 1.0E-25) had average alignment of 7.6% (range of percentage hit length: 1.1-27.6%) and the average length of the aligned region was about 45 bp (range of hit length: 19–157 bp; Additional file 1). These low homology hits may have occurred by chance as suggested by their poor E-values and relatively short alignments between the hit (assembled contig sequence) and the Sanger sequence. Moreover, several of the Sanger sequences are not known to be expressed in gill tissues such as those that encode gonadal specific proteins and they had hits with very low coverage (Additional file 1). This is apparent in that 67% (8/12) of the hits with poor E-value had average coverage less than 20-fold which is very low when compared to house-keeping or abundantly expressed genes such as *beta-actin*, *ribosomal protein L18*, *protein kinase C receptor*, *nucleoside diphosphate kinase* which had average coverage greater than 10,000-fold based on our NGS data. Transcripts associated with molecular transport such as *nka alpha subunit 3*, *nkcc1a* cotransporter, and *chloride-like channel 5*, which are known to express in the gill tissues had average coverage of hundreds to thousands of fold. Taken together, the analysis further indicates specificity and representation of transcripts of various abundances within the assembled FW&SW gill transcriptome.

### *In silico*annotation of combined FW&SW gill transcriptome

To facilitate functional annotation of the combined FW&SW gill transcriptome, we performed BLASTX using assembled contigs having 20-fold or greater average coverage because low coverage contigs are more susceptible to noise and therefore increases the likelihood of spurious assembly. The selected 77059 contigs that have 20-fold or greater average coverage were BLASTX to *O. niloticus* (Nile tilapia) protein database, *Danio rerio* (zebrafish) protein database and NCBI *Homo sapiens* (human) non-redundant (nr) protein database for annotation and comparison purpose (refer to Methods for the web-links to these databases). The *O. niloticus* database was chosen because it is the species most closely related to *O. mossambicus*, and *D. rerio* database was chosen for comparison purpose with a fish model that is more distantly related, while human database is chosen for its extensive annotation useful for knowledge-based data mining. The findings showed that BLASTX to *O. niloticus* database consistently yielded the highest number of mapped contigs and unique identifiers, and this was followed by zebrafish database and human nr database (Table 1). Species with closer phylogenetic relationship with *O. mossambicus* such as *O. niloticus*, followed by *D. rerio*, would yield higher number of contigs and unique identifiers with higher homology hits compared to human that is of greater phylogenetic distance. The number of unique identifiers that were mapped to human and zebrafish databases is comparable at E-value ≤ 1.0E-50, however, the number of unique identifiers mapped to human exceeded that of zebrafish at E-value between 1.0E-50 to 1.0E-05. This is due to the higher number of unique identifiers that are of lower homology as a result of phylogenetic distance when BLASTX to human database. Moreover, the human nr database having extensively annotated would have a larger number of sequences, therefore providing more opportunity for lower homology hits. The number of unique identifiers estimated in the FW&SW gill transcriptome at E-value ≤ 1.0E-50 is within the range, if not too far from the range of 11000–16000 protein-coding genes expressed in transcriptomes of various human and mouse tissues [16] although direct comparison is not possible due to different databases and data processing approach used. At E-value < 1.0E-50 and E-value < 1.0E-20, the *O. mossambicus* FW&SW gill transcriptome has contigs that mapped to 36.2% and 40.7%, respectively, of the unique identifiers found in *O. niloticus* protein database. This suggests that the *O. mossambicus* FW&SW gill transcriptome may be useful for aiding the annotation of the *O. niloticus* databases using high homology sequences (E-value < 1.0E-50). Since tilapia identifiers are not supported by existing knowledge-based data mining software, and the human database is perhaps the best annotated with research data and supported by a wide range of software, human homologs were used to facilitate subsequent functional annotation and analyses. We adopted a more conservative E-value ≤1.0E-50 to gain greater confidence of the human homologs used in the subsequent functional analyses. Approximately 99.4% and 86.2% of the contigs that were mapped to human homologs at E-value ≤1.0E-50, have equivalent E-value ≤1.0E-50 and E-value ≤1.0E-100, respectively, when BLASTX to *O. niloticus* protein database. This shows that the assembled contigs selected for functional analysis are of high homology to protein sequences found in the human and *O. niloticus* databases.

**Table 1:** **BLASTX of combined FW&SW assembled contigs mapped to** ***Oreochromis niloticus*** **,** ***Danio rerio*** **and** ***Homo sapiens*** **protein databases**

Database	BLASTX E-value range	Number of mapped contigs (Percentage of total selected contigs)*	Number of mapped unique identifier in protein database (Percentage of sequences in respective database)**
*Oreochromis niloticus* (NCBI Nile tilapia protein database release 101 containing 45443 sequences)**	0	9845 (12.8%)	8830 (19.4%)
	≤1E-100	15733 (20.4%)	12946 (28.5%)
	≤1E-50	23556 (30.6%)	16433 (36.2%)
	≤1E-20	32541 (42.2%)	18474 (40.7%)
	≤1E-05	38925 (50.5%)	19510 (42.9%)
*Danio rerio* (NCBI zebrafish protein database release 103 containing 42940 sequences)**	0	5667 (7.35%)	4989 (11.6%)
	≤1E-100	10410 (13.51%)	8594 (20.0%)
	≤1E-50	16452 (21.35%)	12133 (28.3%)
	≤1E-20	24150 (31.34%)	14779 (34.4%)
	≤1E-05	31775 (41.23%)	16397 (38.2%)
*Homo sapiens* (Direct BLASTX to the NCBI human non-redundant database)**	0	4418 (5.7%)	4063
	≤1E-100	8583 (11.1%)	7796
	≤1E-50	14018 (18.2%)	12317
	≤1E-20	20785 (27.0%)	17022
	≤1E-05	28446 (36.9%)	21075

*Total selected contigs (average coverage ≥20) =77062. Percentage of total selected contigs = (number of contigs/77062) x 100%.

**Percentage of Nile tilapia protein database = (Number of unique identifiers/45443) x 100%. Percentage of zebrafish protein database = (Number of unique identifiers/42940) x 100%. Direct BLASTX to the NCBI human non-redundant database were used and information on exact number of sequence is not available, therefore percentage of human nr database is not determined.

### Functional annotation analysis of transcriptomic differences between FW gill and SW gill

**Figure 3:**
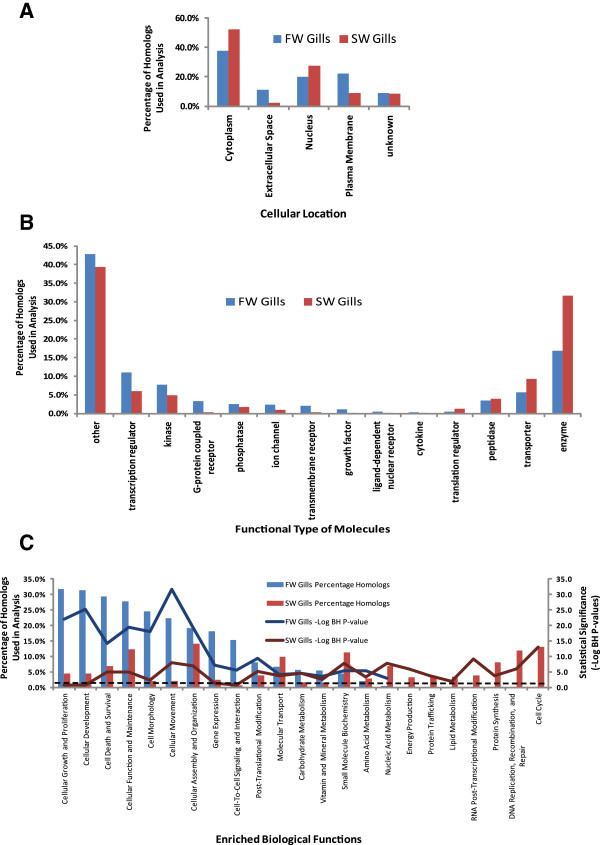
**Functional annotation analyses of the combined FW&SW gill transcriptome of** ***Oreochromis mossambicus*** **. (A)** Distribution (percentage of homologs used in analysis) of the putative human homolog encoded proteins within different sub-cellular locations. **(B)** Distribution of the putative human homolog encoded proteins according to their functional type. **(C)** Biological functions that were significantly [Benjamini-Hochberg (BH) adjusted *P-value* < 0.05] enriched with putative human homolog proteins encoded by the assembled contiq sequences used in the analysis. The dotted line refers to –Log_10_ BH *P-value* = 1.301 which is equivalent BH *P-value* = 0.05 and all the solid lines above the dotted lines have (BH) adjusted *P-value* < 0.05.

**Figure 4:**
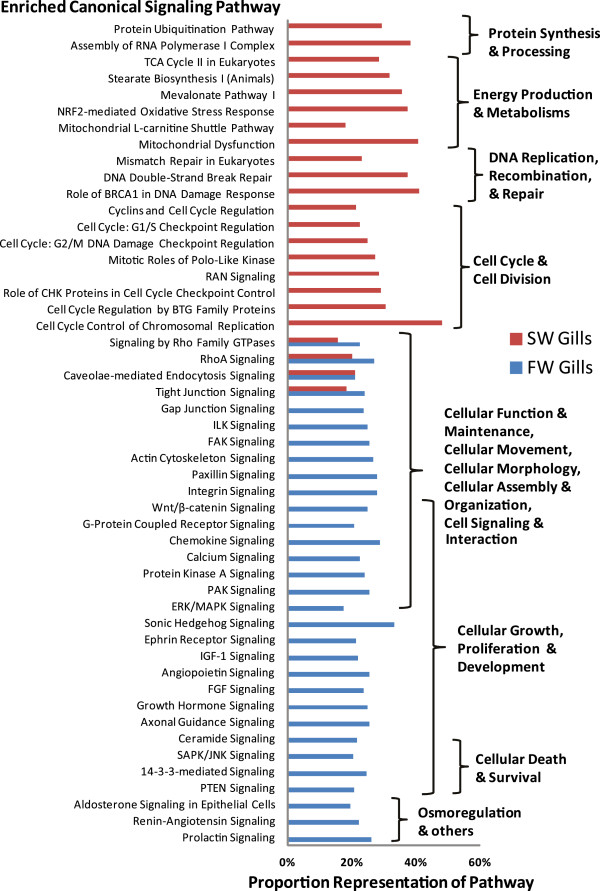
**Canonical signaling pathways that were significantly (Benjamini-Hochberg adjusted P-value < 0.05) enriched with putative human homolog proteins encoded by the assembled contiq sequences used in the analysis.** Proportion (%) of the signaling pathway being represented by the human homolog encoded proteins is been indicated on the x-axis. Refer to Additional file for complete list.

To determine the relative transcriptomic differences in FW gill and SW gill, the reads from each library were mapped with high stringency back to the combined FW&SW gill transcriptome. Approximately, 71.2% and 71.5% of the reads from respective FW and SW libraries were mapped back to the combined FW&SW gill transcriptome. The read counts of FW and SW libraries for each contig were expressed in RPKM values and the expression ratio (SW RPKM/FW RPKM or FW RPKM/SW RPKM) was estimated for each contig (see Methods). Contigs which have E-value ≤ 1.0E-50 when BLASTX to human nr database and average coverage ≥ 20-fold as well as 1.5-fold difference in expression value (RPKM) between SW and FW gills were considered differentially expressed contigs that have high homology to a known human gene. Using these criteria, we obtained 3098 and 2977 human homolog identifiers that were differentially expressed in FW and SW gills, respectively (Additional file 2 and Additional file 3). The human homologs were then used for knowledge-based data mining via Ingenuity Pathway Analysis (IPA) software and the findings are summarized in Figures 3 and 4.

The annotation for cellular localization and known functions of the human homologs indicates that FW gills has relatively larger proportion of transcripts encoding proteins that are found on the plasma membrane and extracellular space when compared to SW gills which has greater abundance of transcripts encoding nuclear and cytoplasmic proteins (Figure 3A). Further analysis revealed that FW gills has markedly higher proportion of transcripts encoding G-protein coupled receptors (13-fold higher), transmembrane receptors (8-fold higher), growth factors (8-fold higher), cytokines (6-fold higher), ion channels (2.5-fold higher), ligand-dependent nuclear receptor (2.5-fold higher) when compared to SW gills which in turn has more abundant transcripts encoding translation regulators (3-fold higher), metabolic enzymes (2-fold higher) and transporters (1.5-fold higher) (Figure 3B). Analysis on the biological functions suggests that both FW and SW gills were significantly (Fisher’s Exact test with Benjamini and Hochberg adjusted P-value < 0.05) enriched with transcripts encoding proteins associated with Cellular Function and Maintenance, Cellular Movement, Cellular Morphology, Cellular Assembly and Organization, Cell Death and Survival, Post-Translational Modification, Molecular Transport, Carbohydrate, Vitamin and Mineral Metabolisms (Figure 3C). The percentage of transcripts encoding proteins associated with these biological functions was 2 to 12-fold higher in FW gills compared to SW gills. In addition, FW gills alone were significantly enriched with transcripts encoding proteins associated with Cellular Growth and Proliferation, Cellular Development, Cell-To-Cell Signaling and Interaction. In contrast, SW gills alone were significantly enriched with transcripts encoding proteins associated with Cell Cycle, DNA Replication, Recombination, and Repair, Protein Synthesis, RNA Post-Transcriptional Modification, Protein Trafficking, Energy Production, Lipid, Nucleic Acid and Amino Acid metabolisms. The analysis provides us a differential transcriptomic perspective that suggests different type of encoded proteins and their associated biological functions were enriched based on the differences in relative abundance of transcripts between FW and SW gills. Even so, the enrichment of a biological function in one condition merely suggests greater relative abundance of transcripts for the encoded proteins associated with the biological function and it does not necessary mean the absence of this biological function operating in the condition that is not enriched.

Deeper analysis revealed underlying canonical signaling pathways that might be driving these biological functions in FW and SW gills (Figure 4). It was found that signaling pathways involving RhoA and other Rho Family GTPases, Tight Junction Signaling and Caveolae-mediated endocytosis were significantly (Fisher’s Exact test with Benjamini and Hochberg adjusted P-value < 0.05) enriched in both FW and SW gills. These signaling pathways are known to be involved in Cellular Function and Maintenance, Cellular Movement and Morphology, Cellular Assembly and Organization identified in Figure 3C. More importantly, RhoA signaling by Rho GTPases regulates actin polymerization [17] which in turn affects tight junction via caveolae-mediated endocytosis and alters the intercellular permeability of the epithelial layer [18]. The enrichment of these signaling pathways in both FW and SW gills underscored their importance in regulating paracellular permeability of the gills. It has been proposed that tight junctions affect paracellular permeability to sodium ions in SW and FW gills [19] and the expression of tight junction proteins such as claudins was altered in FW and SW gills [20, 21]. In agreement with Tipsmark et al. [20], our data showed that human homolog *claudin 4* was more abundant in FW gills than SW gills. Moreover, our findings suggest different sets of human homologs including claudins and tight junctions are acting in these signaling pathways to bring about the differences in paracellular permeability of the gills acclimated in FW and SW environments.

In FW environment, the gills are subjected to hypo-osmotic stress with excessive water gain and salt loss. In response to the hypo- osmotic stress, signaling pathways related cell morphology and cell volume regulation will be up-regulated to reduce water gain and adjust cell volume. Moreover, signaling pathways involved in the remodeling of the cellular landscape by increasing ionocytes with ability to sequester and reabsorb salt lost to the low ionic environment are anticipated to be up-regulated. Consequently, signaling pathways that were enriched only in FW gills involved actin cytoskeleton, integrin, gap junction, ILK, paxillin, FAK, Wnt/β-catenin, and also various cell signaling pathways such as those involving PAK, ERK/MAPK, protein kinase A, calcium, SAPK/JNK, and G-protein coupled receptor (Figure 4). These signaling pathways could play important roles in cellular adhesion, movement and morphology in response to osmotic stress as had been documented in other cell types [22]. In agreement with previous studies [2, 23, 24], our analysis indicated that transcripts associated with MAPK signaling, an important pathway in osmotic stress response, abound in FW gill and they were down-regulated in SW gill of tilapia. Interestingly, it has been proposed that osmotic stress may act as signals, either directly or indirectly via cell volume changes, that could induce cell proliferation and differentiation as well as programmed cell death [9, 22, 25, 26]. Signaling pathways associated with cellular growth, proliferation and development such as axonal guidance, FGF, growth hormone, angiopoeitin, IGF-1, ephrin receptor and sonic hedgehog, were enriched in FW gills only (Figure 4). In determining cell death and survival are PTEN signaling, 14-3-3-mediated signaling, SAPK/JNK signaling which were also enriched in FW Gills only. It is noteworthy that in the euryhaline killifish (*Fundulus heteroclitus*), the expression of *14-3-3a* isoform and its protein had been demonstrated to be more abundant in FW after 24 h or 48 h transfer from SW [27]. Consistent with their findings [27], the present study showed that transcripts associated with 14-3-3 signaling pathway were more abundant in FW when compared to SW even after long term acclimation. Besides cell death and proliferation, 14-3-3 proteins have been implicated in iono-regulation involving H^+^-ATPase, NKA, and chloride channels [27, 28]. The signaling pathway associated with prolactin is known to be involved in FW osmoregulation [5, 29]. It has been observed that both prolactin receptor 1 and 2 are expressed at higher levels in the gills of *O. mossambicus* acclimated long term in FW compared to SW [30]. Prolactin has been shown to restore the expression of branchial *NCC* mRNA levels and its encoded protein in immuno-histologically stained ionocytes of hypophysectomized *O. mossambicus* suggesting its essential role in the recruitment of branchial NCC-expressing ionocytes during FW acclimation [31]. These studies corroborated with the enriched prolactin signaling in gills of long-term FW acclimated *O. mossambicus* observed in the present study. Although their precise mechanistic roles in gill epithelial cells are less clear, the renin-angiotensin and ‘aldosterone-like’ signaling pathways enriched in FW gill are associated with electrolyte and fluid homeostasis hence related to volume regulation in fish as observed in higher vertebrate [5, 29, 32]. The various signaling pathways represented in the FW gill suggest multiple signals acting in concert via osmotic stress signaling to balance cell proliferation, growth and development with cell death and survival. This is in agreement with recent studies reporting different signaling molecules involved in the development and functional regulation of ionocytes in FW species such as zebrafish [33–35] and medaka [36].

In SW environment, fish needs to replace water loss to the hyper-osmotic environment by imbibing salt water and absorbing it via the intestinal tract, whereby water is retained in the body and excess salt is extruded via the gill. Consequently, the gills need to increase the amount of specialized ionocytes enriched with mitochondria to actively extrude the excess salt against concentration gradient. In coping with hyper-osmotic stress and the energetically demanding salt extrusion, the increased cellular metabolism and mitochondrial activities could lead to oxidative stress resulting in cellular damage and turnover. In turn, signaling for DNA replication and repair, protein synthesis and degradation as well as cell cycle regulation are increased. Therefore, it was found that canonical signaling pathways that were enriched only in SW gill are mainly associated with the following categories: cell cycle and cell division; DNA replication, recombination and repair; metabolism and energy production; protein synthesis and degradation (Figure 4). Our findings corroborated with previous reports on remodeling of cellular landscape by increasing ionocytes in tilapia gills under salinity challenge or hyperosmostic stress [37–39]. These ionocytes are responsible for active ion transport in gills which require increase cellular metabolism and energy hence they are mitochondrion-rich [6]. This likely contributed to the enrichment of canonical pathway associated with mitochondria activities, TCA cycle and lipid (mevalonate and stearate) metabolism. The increase of ionocytes is a result of increase mitotic activity to increase its cellular capacity to iono-regulate [38, 39]. This further explains the enrichment of many canonical pathways associated with cell cycle in SW gill such as cell cycle control of chromosomal replication, cell cycle regulation by BTG family proteins, RAN signaling, cyclins and cell cycle regulation, mitotic roles of polo-like kinase, cell cycle: G1/S checkpoint regulation and cell cycle: G2/M DNA damage checkpoint regulation. Moreover, hyperosmolality has been reported to cause DNA damage in the form of DNA double-strand breaks and oxidative base modification that are recognized by the cellular genome integrity surveillance machinery [40, 41]. This is in agreement with the enrichment of canonical pathways such as DNA double-strand break repair, mismatch repair in eukaryotes and role of BRCA1 in DNA damage response in SW gill. Besides DNA damage, hyperosmolality also causes oxidative stress and protein damage [41, 42] which could have resulted in the enrichment of canonical pathways such as mitochondria dysfunction, NRF2-mediated oxidative stress response and protein ubiquitination. This corroborates with studies reporting that the turnover rate of ionocytes in gills under salinity challenge is faster than in FW gill [7, 38, 43] which further explains the high level of transcripts associated with cell cycle, mitosis, DNA replication and repair, as well as mitochondrial energy metabolism. These enriched signaling pathways represent the concerted efforts of the SW gill to remodel and maintain the cellular landscape to better iono-regulate in the hyperosmotic environment.

Taken together, the analysis revealed a myriad of signaling pathways in FW gill coordinating the balance of cellular maintenance, volume/size adjustment, adhesion, morphology, renewal, growth and development under hypo-osmotic stress while the SW gill abounded with transcripts encoding proteins associated with signaling pathways that drive and maintain the cellular remodelled landscape with increased ionocytes to cope with the higher cellular turn-over rate and iono-regulatory demands under hyper-osmostic stress. The differential transcriptomic analyses revealed for the first time the transcriptomic differences between the gills of tilapia acclimated in FW and SW, hence expanding our view from a relatively few well-studied molecules to a plethora of transcripts in association with various biological functions and specific canonical signaling pathways. Since the transcript homologs used in the analysis are of high homology (E-value < 1E-50) to human, it can be assumed with confidence that these signaling pathways are conserved and are actively operating in the fish gills.

### Transcripts associated with inorganic ion channels and transporters in FW and SW gills

**Figure 5:**
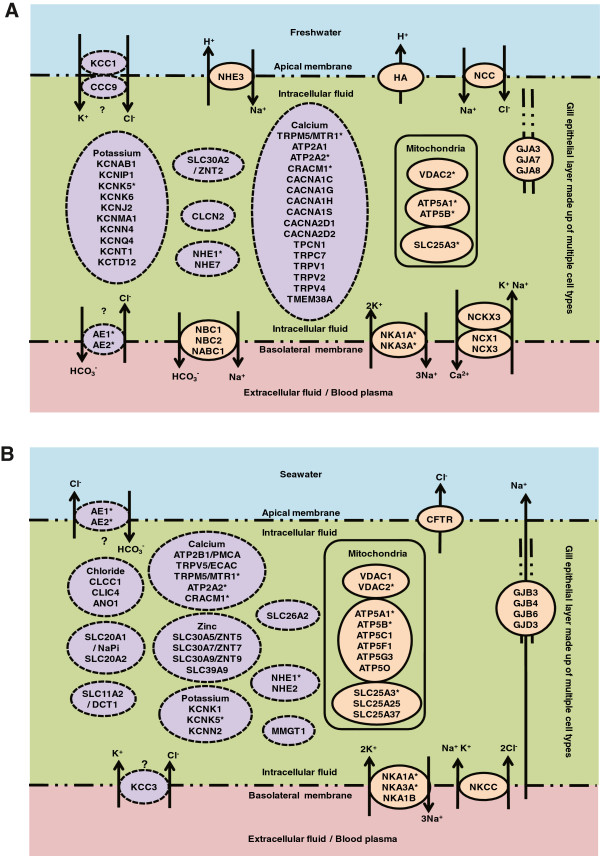
**Schematic view of selected inorganic ion channels and transporters encoded by genes identified as differentially expressed in gill epithelia of tilapia** ***Oreochromis mossambicus*** **acclimated in (A) freshwater (FW) or (B) seawater (SW).** In actual, the simplified epithelial layer is made up of various cell types (not shown here) and the genes encoding these proteins are expressed in different combinations in different cell types. Some of these proteins may be found in different subcellular localizations in different cell types or in different species. Ion channels or transporters where their subcellular localization are well established or have been reported in tilapia are represented in orange oval with solid line. Ion channels or transporters that are lesser known in fish are represented in purple oval with dotted line. Asterisks ‘*’ refer to putative isoforms arose from unique contigs that were mapped to identifiers assigned with the same gene symbol and appeared to be differentially expressed in both FW and SW gills. ‘?’ indicates unconfirmed or potential subcellular localization based on its putative identity. For more details refer to the text and Additional file 4.

In our interest to identify additional transcripts encoding ion channels and transporters that might be differentially expressed in the FW and SW gills, we selected human homologs that had been functionally annotated as ‘ion channel’ or ‘transporter’ by IPA. From the list, we further confirmed and selected out those that are associated with inorganic ion movement based on functional description in Entrez/NCBI Gene and GeneCard databases (see Methods). In total, we identified 59 and 47 human homologs in FW and SW gills, respectively (Additional file 4). Among these ion channels and transporters, 14 of them were likely to have isoforms in FW and SW as they have different identifiers when BLASTX to NCBI Human non-redundant database suggesting minor sequence difference between the isoforms although they were mapped to similar human gene symbols by IPA. Our list identified ion channels and transporters that are well studied, and also those that are lesser known, for their iono-regulatory roles in the gill. For illustration purpose, we have presented all the identified ion channels and transporters of human homologs in simplified figures (Figure 5A and B) although in actual these genes and their encoded proteins are expressed in different combinations in different cell types as reported in gill epithelia of FW and/or SW teleosts. Ion channels or transporters where their subcellular localization are well established or have been reported in tilapia are shown in Figure 5 although some of these proteins may be found in different subcellular localizations when in different cell types or in different species as discussed below.

In low-ionic and hypo-osmotic FW, the gill needs to reduce salt loss and water gain. To reduce salt loss, ion transporters that can aid in sequestering and reabsorbing salt from the low ionic FW environment are expressed abundantly in the FW-acclimated gills. Several homologs of key ion transporters that aid in reducing salt loss such as NCC and isoforms of NKA, NHE, NBC, AE, were identified as over-expressed in our FW gill transcriptome which confirmed previous reports [5, 7, 44–50] that these molecules were abundant in the gills of FW-acclimated fish (Figure 5A). Uptake of Na^+^/Cl^-^ from the environment, including ions diffusively lost from adjacent pavement cells, into the gill ionocytes is partly achieved by NCC and NHE located in the apical membrane of different cell types [44, 45]. Recent evidence suggests that NHE together with ammonia-conducting Rh proteins, especially Rhcg, located apically play an important role in Na^+^ uptake in FW environment and may couple with ammonia excretion [46]. Transcript encoding Rhbg and Rhcg homologs were detected more abundant in FW-acclimated gills (see Additional file 4). Additionally, apical H^+^-ATPase immuno-localized in pavement cells of tilapia gills has been implicated in acid secretion and the generation of apical membrane potential to facilitate Na^+^ uptake by sodium channel [47]. Interestingly, basolateral H^+^-ATPase has been detected in ionocytes of killifish gills, presumably working together with NKA to generate a steeper electrical gradient for absorption of Na^+^ from the low Na^+^ environment [48]. The different subcellular localizations may be due to the diverse distributions and functions of H-ATPase in different cell types and different species. NCC brings in Na^+^ and Cl^-^ by utilizing the low intracellular sodium gradient generated by the NKAs which facilitates the basolateral exit Na^+^ into the blood plasma [7]. Meanwhile, NBCs reported to be located basolaterally in NCC-positive ionocytes [49] could co-transport Na^+^ and HCO_3_^-^ which in turn will aid exit of Na^+^ together with basolateral base [HCO_3_^-^] efflux that complements apical acid secretion in regulating intracellular pH. We detected up-regulation of a *CLCN2* homolog which may facilitate basolateral exit of Cl^-^ as proposed for some of these chloride-like channels in gills of FW-acclimated tilapia [50]. In addition, we have detected two FW-isoforms of anion exchanger (AE) homologs, SLC4A1 and SLC4A2 (see Additional file 4), although their localization remains unclear. The presence of apical AE to aid Cl^-^ uptake/HCO_3_^-^ base secretion had been proposed [5, 47] but the specificity of AE1 detection was questioned [51] and basolateral AE has been counter-proposed [7]. We also detected isoforms of NCX and NKCX homologs which are known to function as basolateral cation exchangers by removing Ca^2+^ out of the cell in exchange of 2 Na^+^ alone or with 1Na^+^:1 K^+^ into the cell. Immunohistochemical staining of FW-acclimated tilapia gill had co-localized apical NCC with basolateral NKA and NBC in proposed type II ionocytes [44], as well as apical NHE with basolateral NKCC1 and NKA in proposed type III ionocytes [52, 53].

In SW environment, salt gained from the hyperosmotic environment need to be extruded from the gills hence ion transporters that facilitate salt extrusion are anticipated to be over-expressed in SW-acclimated gill. Indeed, we were able to detect in abundance the transcript sequences for the three major ion-transporters NKA, NKCC1 and CFTR that are known to be involved in active salt extrusion (Figure 5B). A well-accepted model for the salt extrusion involved basolateral NKA and NKCC1 in specific ionocyte whereby NKA transports 3 Na^+^ outward in exchange for 2 K^+^ inward thus creating a low Na^+^ and an electro-negative charged intracellular environment that is used by NKCC1 to transport Na^+^, K^+^ and 2 Cl^-^ into the cell [5, 7]. The Cl^-^ leaves the cell via the apical CFTR which generates a positive trans-epithelial electrical potential (TEP), while the Na^+^ is transported back into the plasma via the basolateral NKA, which the Na^+^ then leaves via a paracellular tight junction pathway driven by the positive TEP. Not surprising, we have detected abundance of transcript sequences for several ATP-synthases and transporters operating in the mitochondrion since the ionocytes are enriched with mitochondria to support the active salt extrusion and the number of ionocytes is increased in SW gill. Immunohistochemical staining that co-localized apical CFTR with basolateral NKA and NKCC1 have been demonstrated in ionocytes corresponding to the proposed type IV (the SW type) ionocyte on the yolk sac embryos of Mozambique tilapia acclimated in SW [44].

We have also identified numerous transcript sequences of putative transporters/channels for K^+^, Ca^2+^, Cl^-^, Zn^2+^ as well as inorganic phosphates and sulfate that are not well-studied or lesser known for ion regulation in fish gill (Figure 5A and B). The many transcript sequences involved in ion-regulation of Ca^2+^, K^+^ and Cl^-^ may have roles in cell volume regulation. Efflux of K^+^ and Cl^-^ are required for regulatory volume decrease hence important for reducing cell swelling in a hypotonic environment such as gill epithelia in FW [3, 22]. Influx of Ca^2+^ is also involved in regulatory volume decrease since several K^+^ and Cl^-^ channels are activated by Ca^2+^, and similarly Ca^2+^ act as secondary messenger in many intracellular processes via calcium signaling [2] as enriched in FW gill transcriptome. In SW gill, increase in transcript sequences for members of slc30 (ZnT) members may suggest flux of Zn^2+^ from cytosol out of the cell or into organelles necessary for many catalytic and structural roles involved in transcription regulation, nucleic acid synthesis, protein trafficking and signaling [54]. Interestingly, we detected different transcript abundance for putative gap junction (GJ) proteins where alpha forms (*GJA3, GJA7, GJA8* homologs) were more abundant in FW gill while beta forms (*GJB3, GJB4, GJB6* homologs) were more abundant in SW gill. GJ proteins are intercellular channels that connect neighbouring cells permitting ions and metabolites movement between them, and possibly play a role in tissue regeneration and cellular remodeling [55, 56]. The different abundance of GJ protein forms may be due to the different populations of ionocytes found in the FW and SW gills using different GJ proteins to interconnect with their neighbouring cells such as accessory, pavement and glycogen-rich cells. Recently, the presence of different population of ionocytes, possibly including maturing ionocytes or accessory cells as well as ‘undifferentiated cells’ attaching to mature ionocytes, was detected in the tilapia gills using fluorescent staining with anti-NKA, anti-BrdU and DAPI [39]. The study was on recruitment and degeneration of different population of BrdU-labelled branchial ionocytes in Mozambique tilapia transferred from FW to 70% SW and it reported a population of ionocyte described as ‘single BrdU-labelled mitochondrion-rich cells (MRC)’ which increased in both FW and 70% SW, and another population described as ‘BrdU-labelled MRC-complexes’ which increased predominantly in 70% SW [39]. Taken together, the present findings indicate that a plethora of transcripts encoding ion channels and transporters are likely to be expressed in different cell types including ionocytes and non-ionocytes that populate the gills of FW and/or SW tilapia.

### Targeted gene expression in gill subjected to hypersalinity challenges

**Figure 6:**
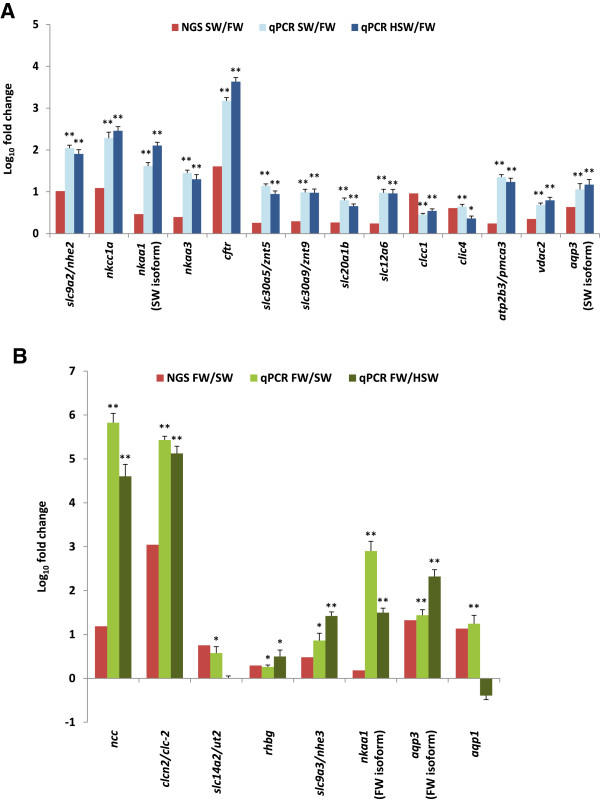
**NGS data and quantitative real-time PCR of representative genes encoding inorganic ion channel or transporters that were identified as differentially expressed i.e. (A) abundant in freshwater (FW) gill or (B) abundant in seawater (SW) gill of** ***Oreochromis mossambicus*** **.** Data that assume normality and homogeneity of variance were subjected to ANOVA followed by Tukey-Kramer post-hoc test. Data that do not assume normality or homogeneity of variance were subjected to Kruskal Wallis followed by Mann–Whitney *U* test and Bonferonni correction for multiple comparisons. **indicates *P-value* < 0.01 while *indicates *P-value* < 0.05 when SW and HSW are compared to FW for the same gene. *N* = 6–7 replicates.

**Figure 7:**
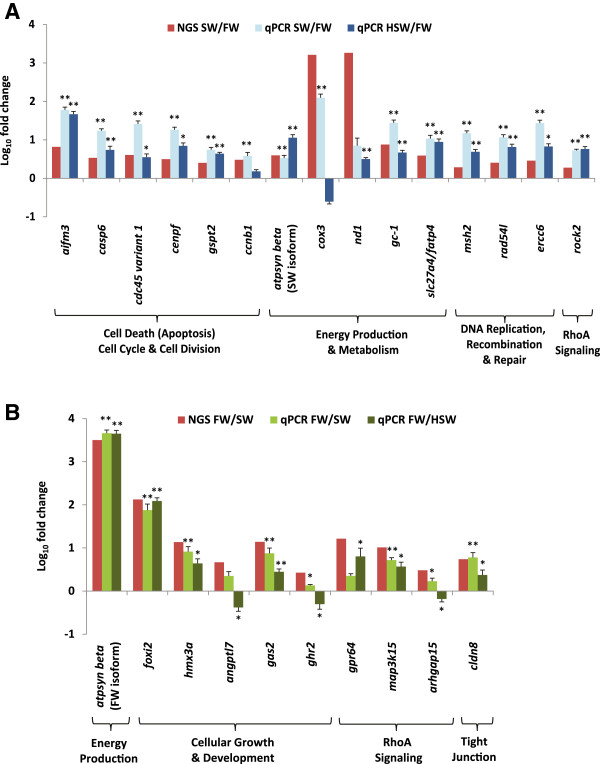
**NGS data and quantitative real-time PCR of representative genes associated with canonical signaling pathways or biological functions that were identified as differentially expressed i.e. (A) abundant in freshwater (FW) gill or (B) abundant in seawater (SW) gill of** ***Oreochromis mossambicus*** **.** Data that assume normality and homogeneity of variance were subjected to ANOVA followed by Tukey-Kramer post-hoc test. Data that do not assume normality or homogeneity of variance were subjected to Kruskal Wallis followed by Mann–Whitney *U* test and Bonferonni correction for multiple comparisons. **indicates *P-value* < 0.01 while *indicates *P-value* < 0.05 when SW and HSW are compared to FW for the same gene. *N* = 6–7 replicates.

We performed additional experiments using a new batch of fish acclimated to FW (0 ppt), SW (30 ppt) and hypersaline water (HSW; 70 ppt) to validate the NGS data and to further investigate how representative genes behave in gill subjected to HSW challenge. The Mozambique tilapia is among few fish species that is able to cope with the tremendous iono-regulatory challenges under HSW. If the genes involved in iono-osmoregulation or responded to salinity challenge via up-regulation (i.e. high SW/FW ratio) or down-regulation (i.e. high FW/SW ratio) remained so, if not increased in magnitude, when the fish is subjected to hypersalinity challenge, it will show that these gene expressions are also necessary for iono-osmoregulation during hypersalinity challenge notwithstanding the short-term acclimation in HSW. We performed targeted gene expression analysis using real-time quantitative PCR on representative genes associated with biological processes/pathways identified earlier including iono-osmoregulation, cell cycle/death, DNA replication, recombination and repair, growth and development, energy and metabolism, and intracellular signaling. The relative gene expression levels as determined by real-time PCR as compared to the NGS data are summarized in Figures 6 and 7 (see Additional file 5 for primers information). Overall, the real-time PCR data for SW and FW gills were in good concordance with the NGS data although the PCR data captured greater magnitude in relative fold difference of gene expression. Among the 47 genes investigated (29 SW-abundant and 18 FW-abundant), 38 genes (~81% of total genes investigated) showed significant (*P* < 0.05) deregulation in the same expression direction for SW- and HSW-gills when compared to FW-gills confirming that majority of these genes are responsive to salinity challenge (Figures 6 and 7). The validation of these representative genes provide further evidence that the associated biological processes and pathways were indeed deregulated in FW, SW and HSW gills.

The many similar gene-expression trends in HSW and SW when compared to the same FW control group shows that the expression of these genes are highly robust and necessary for iono-osmoregulation during hypersalinity challenge notwithstanding the different acclimation state. This is clearly evident for *nkcc1a*, *nkaa1*, *nkaa3* and *cftr* which are well established in their osmoregulatory roles during salinity challenge. The increase in *atpsyn beta* transcripts in SW and HSW represents increase in cellular energy production for active salt extrusion. In order to drive Na^+^ to exit from the plasma to the external environment, a trans-epithelial potential (TEP) that is greater than the equilibrium potential of Na^+^ is (*E*_*Na*_) is required. As internal plasma Na^+^ is maintained constant while external Na^+^ increased with salinity, *E*_*Na*_ will rise rapidly. With the increase of salt load into the fish due to the drinking of salt water to compensate water loss to the hyperosmostic environment, increase in basolateral NKA and apical CFTR activities are required to actively generate sufficient positive TEP > *E*_*Na*_ in order to drive Na^+^ against concentration gradient from the plasma into the hypersaline environment via leaky paracellular tight junctions between the ionocyte and accessory cell. The increase of *nkaa1, cftr,* and *atpsyn beta* transcripts can be contributed by direct increase in expression per ionocyte as well as increase in the number of ionocytes [39, 57]. Since further uptake of Na^+^ can be detrimental, this necessitated further down-regulation of the FW-abundant NHE3 which facilitates apical Na^+^ uptake/acid secretion and appeared to be present at low levels in SW-gill [52]. Likewise, *aqp3* (FW-isoform) which was proposed to be involved in osmoreception and regulation of cell volume by facilitating basolateral water flux between intracellular ionocyte and plasma [58], was further down-regulated likely to reduce water permeability and prevent excessive shrinkage of cells when plasma osmolality rises in HSW. We have also sequenced and detected a putative *aqp3-like* (SW-isoform) that was up-regulated in SW and HSW when compared to FW. This is the first report of *aqp3* (SW-isoform) up-regulation in SW and HSW gills although its biological significance is presently unknown. Up-regulation of AQP3 has been proposed to facilitate glycerol transport into skin cell which is a metabolic substrate of central importance for gluconeogenesis and ATP generation [59]. Moreover, glycerol is also a natural water retention osmolyte that could reduce intracellular water loss [60]. Therefore, *aqp3* (SW-isoform) up-regulation may contribute to transporting glycerol as fuel for energy production in the increased number of gill ionocytes and may also help in reducing intracellular water loss in hyperosmotic environment. It is therefore likely that the two isoforms of *aqp3* were expressed in different subcellular localization and different cell types.

There are data suggesting a critical salinity threshold that could induce the onset of osmoregulatory stress in tilapia at 70 ppt and beyond [10, 57, 61]. In a hypersalinity experiment involving California Mozambique tilapia hybrid, plasma osmolality began rising significantly between 65–75 ppt onwards when compared to 35 ppt [61]. This is also corroborated with a significant peak in drinking rate at 75 ppt suggesting excessive water loss hence the need to compensate by increasing drinking rate. The onset of osmoregulatory stress may have caused some of the SW- and FW-abundant genes to have changed in their direction of expression in HSW. It has been proposed that the TEP generated by the increase of basolateral NKA and apical CFTR activities are not sufficient to drive Na^+^ against the increasing chemical gradient as salinity rise beyond 70 ppt, hence resulting in a back-flux of salts via the paracellular tight junction pathway and in the rise of internal Na^+^ concentration until *E*_*Na*_ falls below the TEP in order for Na^+^ extrusion to resume [10]. This may explain the up-regulation of *cldn8* to manage and control the back-flux or influx rate of Na^+^ from HSW into the plasma as it has been shown that claudin 8 can act as a paracellular barrier for Na^+^ back-leakage [62]. In another hypersalinity experiment involving tilapia *Sarotherodon melanotheron*, it was reported that the density and size of gill ionocyte increased significantly between FW to HSW (70 ppt) but the ionocyte density remained constant between HSW at 70 ppt and 90 ppt, although cell size continued to increase with concomitant increase in intensity of NKA staining [57]. The observation suggests that the gill had to rely on increasing cell surface area and NKA activity per cell rather than increasing cell number in order to increase iono-regulatory capacity when presumably maximum cell density is reached at 70 ppt. Therefore cell cycling rate may be reduced to the level sufficient to maintain the cell number and this may account for the down-regulation of some of the SW-abundant genes (*casp6, cdc45 variant 1, ccnb1, msh2, ercc6*) that are associated with cell cycle, DNA replication and repair. The number of cycling and mitotic cells in HSW-gill may also be lowered to conserve energy and channel to meet the energetic demands of iono-osmoregulating in HSW. Sardella et al. [61] reported that the density of mature branchial ionocytes dropped significantly as salinity rose beyond 65 ppt when compared to 35 ppt, presumably as a result of apoptosis as indicated by increasing apoptotic ionocytes although immature ionocytes and accessory cells increased in numbers to replace cell loss. The up-regulation of *aqp1* may be associated with apoptotic volume decrease hence cell shrinkage that is necessary for apoptosis to occur. AQP1 has been reported to facilitate water efflux for dying cell to shrink in order to complete the apoptotic process [63]. Apoptosis may have reduced substantially certain mature ionocytes expressing specific *cox3* isoform in HSW gill. Likewise as part of the cellular remodeling in the HSW gill, up-regulation *angptl7*and *ghr2 arhgap15* are likely associated with cellular renewal, growth, differentiation and recruitment related with the significant increase of immature ionocytes and accessory cells since these genes are known to be affecting cellular growth, development and morphology [64–67]. Taken together, salinity at 70 ppt may be a critical threshold for onset of osmoregulatory stress that induced unexpected but significant changes to the expression direction of several genes in the HSW gill. Moreover, the different acclimation state between SW (long-term acclimation) and HSW (short-term acclimation) could also have contributed to the differences in gene expression trend, suggesting that less differences may be observed if HSW is subjected to similar long-term acclimation. Nevertheless, the expression of many other genes remained unchanged in SW and HSW gills when compared to FW gills while few increased in their magnitude of change in HSW gill when compared to SW gill. The findings confirm that these genes are responsive to salinity challenge.

## Conclusion

Euryhaline plasticity allows individuals to exploit environment with fluctuating salinity for food, shelter and reproduction, be it temporal or long term, thus increasing their chances for survival and reducing competition with stenohaline species. In estuary, euryhaline fish can seek out iso-osmotic environment in brackish water where it is energetically less costly to iono-osmoregulate. On the other hand, the plasticity to switch from hyper-osmoregulation to hypo-osmoregulation or vice versa can be costly because it requires marked changes in the transcriptome, signaling pathways, remodelling of the cellular landscape and energetics as observed in this study. Therefore, it has been proposed that the rarity of euryhaline species in comparison with stenohaline species may reflect the substantial cost of plasticity and therefore unless the benefits of euryhalinity exceed its cost, traits promoting euryhalinity will be lost during evolution hence limiting it only to certain species under special circumstances and selective pressure [1]. From an evolutionary perspective, euryhalinity may be a transitional trait that enables colonization of new adaptive zones hence promoting diversification leading to cladogenesis and eventually resulting in stenohaline taxa [68].

**Figure 8:**
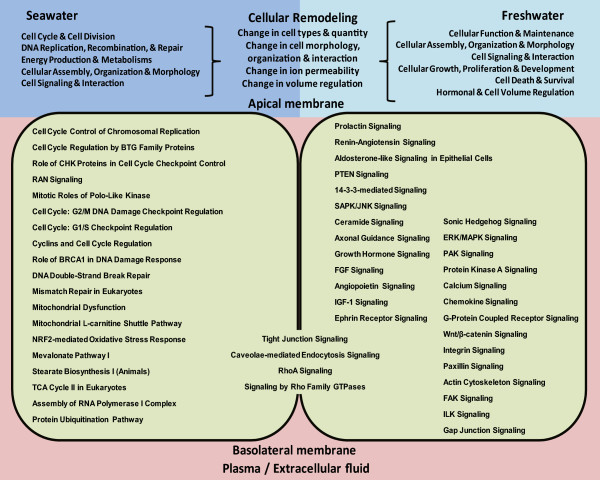
**Summary of canonical signaling pathways and their associated biological functions that effected cellular remodeling and caused changes in cell types and density, cell morphology, organization and interaction as well as ionic permeability and volume regulation in the gills of** ***Oreochromis mossambicus*** **acclimated to freshwater (FW) and seawater (SW).**

We have sequenced and characterized the differential gill transcriptome of Mozambique tilapia acclimated in FW and SW. *In silico* validation indicated that full length and partial sequence of specific transcripts of various abundance and length were represented in the assembled contigs. In addition, we have analyzed and indicated the biological significance of the transcriptomic differences of the FW and SW gills. To our best knowledge, this is the first NGS study that had captured a global perspective of the transcriptomic differences in gills of a euryhaline fish acclimated to FW and SW. The present study has identified over 5000 annotated gene transcripts with high homology (E-value <1.0E-50) to human genes that were differentially expressed in FW and SW gills. In addition, these human homologs were found to be significantly associated with at least 23 biological processes and with over 50 canonical signaling pathways. The association with the many canonical signaling pathways, which has not been reported in relation to gill iono-osmoregulation previously, has provided an expanded and integrated view of how their encoded proteins could co-operate in specific pathways to drive biological functions that effected cellular remodeling in gill under hypo- and hyper-osmotic stresses summarized in Figure 8. These canonical pathways could provide focus for future investigation into specific responsive signaling pathways operating in gill iono-osmoregulation. We have also identified over 100 transcripts that were specifically involved in inorganic ion transporters/channels including several isoforms in which some are known to operate in FW and SW gills while many are lesser known and could be the focus of future studies. We have also validated the expression profile of representative genes in FW and SW gills, as well as investigated their expression in HSW gill. The findings confirmed that many of these genes that responded to salinity challenge would retain their expression profile in HSW gill as in SW gill. However, it was also found that some genes had changed significantly in their expression level or direction in HSW gill when compared to SW gill. This change in gene expression coincided with the critical salinity level of about 70 ppt which is associated with the onset of osmoregulatory stress and further cellular remodeling in response to hypersalinity challenge. In addition, the different acclimation state between SW and HSW could have also contributed to the differences observed. The effects of sex, which is not accounted in this study, cannot be excluded although its effect is likely minimal because all experimental fish, regardless of their sex, have to iono-osmoregulate in order to have survived several weeks in these hypo-osmotic (FW) and hyper-osmotic (SW and HSW) challenging environments. Our findings corroborated with other studies indicating that we have captured many of the molecules and signals at the transcriptome level that are associated with iono-osmoregulation under hypo- and hyper-osmostic stresses. Finally, this study has provided an unprecedented global transcriptomic view of gill iono-osmoregulation since studies were initiated more than 80 years ago; it has expanded our molecular perspective from a relatively few well-studied molecules to a plethora of gene transcripts and a myriad of canonical signaling pathways driving various biological processes that are operating in gills under hypo-osmotic and hyper-osmotic stresses. These findings offer insights and sequence resources that will fuel future studies on gill iono-osmoregulation and cellular remodeling in response to salinity challenge and acclimation.

## Methods

### Fish maintenance and salinity acclimation experiments

Mozambique tilapia *(Oreochromis mossambicus*, total length 10-15 cm; aged about 3–5 months old) maintained in freshwater were obtained from a local fish farm and acclimated to laboratory conditions in dechlorinated tap water (salinity at 0 ppt) for two weeks before experiments. Fish were maintained at a density of about 1 fish/15 L water with gentle aeration under a 12 h/12 h light–dark photoperiod at 25–26°C throughout the experiment. Fish were fed twice daily with commercial fish food (Hikari cichlid bio-gold, Kyorin food Ind. Ltd.) until one day before sampling. Animal procedures adopted in this study were approved by the Institutional Animal Care and Use Committee of the National University of Singapore (IACUC 098/10).

Two sets of salinity experiments were conducted. In the first set of experiment, fish were randomly assigned to two groups. Group I was maintained in dechlorinated tap water (FW; salinity at 0 ppt). Group II were acclimated stepwise with increment of 5 ppt to natural seawater (SW, salinity at 30 ppt) over six transfers (5 ppt, 10 ppt, 15 ppt, 20 ppt, 25 ppt, 30 ppt), allowing two days for acclimation at each transfer of increasing salinity. Group II was then maintained for three weeks in SW until sampling. In the second set of experiment, fish were randomly assigned into 3 groups. Group I was maintained in FW as control group. Group II and III were acclimated stepwise to natural seawater as described above. While group II was maintained at SW until sampling, group III tilapia was acclimated stepwise from SW to hypersaline water (HSW, salinity at 70 ppt) over eight transfers (35 ppt, 40 ppt, 45 ppt, 50 ppt, 55 ppt, 60 ppt, 65 ppt, 70 ppt), with two days allowed for acclimation at each transfer, and finally maintained at 70 ppt for four days before sampling. The entire step-wise acclimation from SW to HSW took about 3 weeks and thereafter fish from FW, SW and HSW were sampled on the same day. Water with salinity below 30 ppt were prepared by mixing dechlorinated tap water with SW, while water above 30 ppt to HSW were prepared by adding sodium chloride (reagent grade >99.8% NaCl, Schedelco, Malaysia) to SW. Salinity was measured using a light refractometer. Fish were anaesthesized with 0.1% (v/v) 2-phenoxyethanol (Sigma-Aldrich, USA) before tissue sampling. The tissue samples were immediately snap-frozen in liquid nitrogen and stored at -80°C until used. Gill tissues were sampled from eight fish for each group in experiments 1 and 2. Gill tissues sampled from experiment 1 were used for next generation sequencing and those sampled from experiment 2 were used for quantitative real-time PCR analysis. Only the highest quality samples were used.

### RNA extraction

Total RNA was extracted using TRIzol® Reagent (Life Technologies Corp., USA) according to the manufacturer’s protocols. Briefly gill tissues were homogenized in 1 ml of TRIzol® reagent, before the homogenate was mixed vigorously with 0.2 ml of chloroform and the aqueous phase was collected after centrifugation. The RNA was precipitated by addition of 0.5 ml of isopropanol and the RNA pellet was centrifuged down and washed twice in 75% ethanol, before the RNA was resuspended in RNase-free water. RNA was quantified using a Nanodrop ND-2000 (Thermo Fisher Scientific Inc., USA) and RNA quality was verified using a 2100 Bioanalyzer with RNA6000 Nano kit (Agilent Technologies Inc., USA).

### Library preparation and sequencing

Equal concentration from four of the highest quality total RNAs (highest RNA integrity RIN > 8.0) extracted from individual fish gills were pooled for FW and SW cDNA libraries preparation. The two cDNA libraries were prepared using TruSeq™ RNA Sample Preparation Kit (Illumina Inc., USA) according to protocol. Briefly, a total of 4 μg of total RNA from each group was diluted with nuclease-free ultrapure water to a final volume of 50 μl. Poly-A containing mRNA were purified using oligo(dT)-attached magnetic beads via two rounds of purification. The purified mRNA is then fragmented using divalent cations at 94°C for 8 minutes and thereafter used for first strand cDNA synthesis using reverse transcriptase Super Script II and random primers. The RNA templates were then removed by RNaseH and the second strand was synthesized using DNA polymerase I to generate double-stranded cDNA fragments. The cDNA fragments were subsequently purified from the reaction mix using Ampure XP beads (Agencourt Bioscience, USA). The 3’ ends were repaired and adenylated before ligated with sequencing adapters. The cDNA fragments were enriched by 15 cycles of PCR amplification using primers that annealed to the ends of the adapters. The size and purity of the cDNA sequencing libraries were determined using a 2100 Bioanalyzer using the DNA-1000 kit (Agilent technologies Inc., USA) and the quantity was estimated using qPCR on a LightCycler® 480 real-time PCR system (Roche Diagnostics, Switzerland). Equal amount of DNA from FW and SW libraries were used for 2 × 101-bp paired-end sequencing on HiSeq 2000 platform using TruSeq SBS Kit v3 – HS (Illumina Inc., USA) and HiSeq Control Software v1.4/RTA v1.2 with Flow Cell v3 following manufacturer’s instruction at the Genome Institute of Singapore. The image analysis and base calling with quality score were performed with CASAVA v1.7 (Illumina Inc. USA) according to manufacturer’s instructions. The raw reads were cleaned by removing adaptor sequences and low quality sequences based on Illumina Pipeline 1.5-1.7 quality scores.

### *De novo*assembly, *in silico*validation and annotation

Paired-end reads were imported into CLC Genomic Workbench 5.0. Reads were trimmed based on Phred scale quality score (*Q15* and below) and failed reads were removed. *De novo* assembly were performed using CLC Genomic Workbench which employed De Bruijn graph-based approach to reconstruct transcripts by assembling contigs from sub-sequences of certain length (k-mer) generated from the reads. Preliminary testing with different k-mers found that higher k-mers assembled shorter contigs but produced better read-mapping while lower k-mers assembled longer contigs but resulted in poorer read-mapping. Subsequently, k-mer = 25 was selected for de novo assembly as it yielded longer N-50 contig length without significant compromise to the read-mapping. Other parameters that were used for the *de novo* assembly included bubble size = 50 (maximum size of bifurbication in the De Bruijn graph that the assembler will resolve as recommended for reads shorter than 110 bp), minimum contig length = 200 and contigs were updated based on the evidence provided by the subsequent read mapping back to *de novo* assembled contigs. Reads mapping back to de novo assembled contigs was performed with local alignment at mismatch score = 2, insertion cost = 3 and deletion cost = 3. Once the optimal alignment of the read is found, based on the costs specified above, a default filtering threshold of length fraction = 0.5 and similarity = 0.9 is applied to determine whether this match is good enough for the read to be included in the output and is further computed as average coverage. Reads from both FW and SW libraries were used to assemble contigs for the combined FW&SW gill transcriptome. To further evaluate the quality of the assembled contigs, we downloaded 60 Sanger-sequenced complete coding sequences of *O. mossambicus* from NCBI Nucleotide database [69] using the search term “*Oreochromis mossambicus* complete cds” and BLASTN these sequences against the combined FW&SW gill transcriptome. The assembled contigs from the combined FW&SW gill transcriptome were BLASTX to *O. niloticus* (Nile tilapia) protein database [70], *Danio rerio* (zebrafish) protein database [71], NCBI non-redundant (nr) database for Human (*Homo sapiens*) via NCBI Blast databases [72] using CLC Genomic Workbench. For downstream functional annotation and pathway analyses, we adopted a more conservative E-value < 1.0E-50 to gain greater confidence of the human homologs used in the subsequent functional analyses.

### RNA-seq mapping and expression analysis

To estimate the relative transcript abundance differences of the assembled contigs in FW and SW gills, the reads from each library were mapped back to the combined FW&SW gill transcriptome using a more stringent setting (minimum length fraction =0.9, minimum similarity fraction =0.8). The read counts that mapped to each assembled contig were subsequently normalized to Reads Per Kilobase per Million reads (RPKM) as the expression value which took into account the length of each contig and the total reads of each library [73]. The expression value in RPKM for each contig in FW and SW gill libraries were used to estimate the expression ratio (SW RPKM/FW RPKM or FW RPKM/SW RPKM) for each contig. The contigs that have E-value < 1.0E-50 and average coverage > 20-fold as well as 1.5-fold difference in expression value (RPKM) between SW and FW gills were considered contigs that have high homology to a known human gene and were differentially expressed.

### Functional annotation and pathway analyses

The human homologs of the assembled contigs that were considered differentially expressed, i.e. abundant in FW gill or abundant in SW gill, were used to mine the human database via Ingenuity Pathway Analysis™ (IPA) software [74]. The software identified the cellular location and the functional type of the encoded products subsequently associated them with biological functions and canonical pathways based on the human knowledge database within IPA. A right-tailed Fisher's Exact test was used to calculate *P*-values in determining the probability that each functional category (biological function or canonical pathway) enriched with the human homologs hence its association with the dataset is due to chance alone. A Benjamini-Hochberg corrected *P-value* < 0.05 is considered significant by the algorithm suggesting that the association is non-random. In our interest to identify transcripts encoding ion channels and transporters that might be differentially expressed in the FW and SW gills, we selected human homologs that had been functionally annotated as ‘ion channel’ or ‘transporter’ by IPA and further confirmed their roles in inorganic ion movement based on the functional description in NCBI Gene database [75] and GeneCard database [76].

### Quantitative real-time PCR

Total RNA were treated with DNase I, amplification grade (Invitrogen) to remove any contaminating genomic DNA. First-strand cDNA was synthesized by reverse-transcription using SuperScript II reverse transcriptase (Invitrogen, USA) with oligo(dT)_20_. All primers for qPCR were designed using Primer3Plus software available online [77]. Primers are listed in Additional file 5. Real-time PCR was performed on a StepOnePlusTM Real-Time PCR system (Applied Biosystems, USA) in a 10 μl reaction using 50 ng cDNA, 200 nM forward and reverse primers and 5 μl of Express SYBR GreenER qPCR supermix with premixed ROX (Invitrogen, USA). Cycling conditions were 95°C for 20s followed by 40 cycles of 95°C for 3 s and 60°C for 30s. Amplification was followed by a melting curve analysis to confirm the specificity of the PCR reaction. The dataset was tested with Shapiro-Wilk test for normality and Levene’s test for homogeneity of variance using SPSS Statistics (IBM, USA). Data that assume normality and homogeneity of variance were subjected to ANOVA followed by Tukey-Kramer post-hoc test. Data that do not assume normality or homogeneity of variance were subjected to Kruskal Wallis followed by Mann–Whitney *U* test and Bonferonni correction for multiple comparisons. A value of *P* < 0.05 was considered to be statistically significant in the analysis. The sample size for each group was *N* = 6-7 replicates.

### Availability of supporting data

The raw reads for the next generation sequencing data have been submitted to NCBI Sequence Read Archive (SRA) [78] with the accession number SRP028106. Other data supporting the results of this article are included within the article and the Additional files 1, 2, 3, 4 and 5.

## Electronic supplementary material

Additional file 1: **Summary BLASTN results for sixty** ***Oreochromis mossambicus*** **full length coding sequences (Sanger sequence from NCBI) which were BLASTN against the combined FW&SW gills assembled contigs.** (XLSX 17 KB)

Additional file 2: **FW-Gill abundant contigs that mapped to human homologs when BLASTX to human non-redundant database (E-value < 1.0E-50).** (XLSX 423 KB)

Additional file 3: **SW-Gill abundant contigs that mapped to human homologs when BLASTX to human non-redundant database (E-value < 1.0E-50).** (XLSX 496 KB)

Additional file 4: **Inorganic ion channels and transporters identified as abundant in freshwater (FW) gill and/or abundant in seawater (SW) gill.** (XLSX 21 KB)

Additional file 5: **Primers and related information for quantitative real-time PCR of selected genes.** (PDF 320 KB)
