# Supplementary material for: Differential transcriptomic analyses revealed genes and signaling pathways involved in iono-osmoregulation and cellular remodeling in the gills of euryhaline Mozambique tilapia, Oreochromis mossambicus
Source: BMC Genomics. 2014 Oct 23;15(1):921. doi: 10.1186/1471-2164-15-921 (PMC4213501; doi:10.1186/1471-2164-15-921)
Supplement: Supplementary file 5 — Additional file 5: Primers and related information for quantitative real-time PCR of selected genes. (PDF 320 KB) [file 12864_2014_6609_MOESM5_ESM.pdf]

Table S2. Primers and related information for quantitative real-time PCR.

Gene symbol	5'-Forward primer-3'	5'-Reverse primer-3'	Amplicon size (bp)	Contig	Description
aifm3	CGCTTCCTGCCAATAAGTCT	AGAGGAGAGAACGGGAAGGT	118	39602	apoptosis-inducing factor 3
angptl7	ACAAGCTCTTCCTTGCCAAC	TGCACAGTCGTCCAAACATT	116	28748	angiopoietin-related protein 7
aqp1	AGGGTTCAAGTGTGCTCCAC	GGAATAGGTCTGGGCAATGA	129	66976	aquaporin-1
aqp3 (FW isoform)	GTTGCTCCGTCCTGGAAGT	TATGCCAGCCGTGTTGTTAG	55	17845	aquaporin-3
aqp3 (SW isoform)	GAACCCATCTCACCCTTGTC	GGCCAGAATGCAAACTATCAG	77	17847	aquaporin-3
arhgap15	CGCAGACAAGAATGGAGTGA	TTCACGCTCACAGAGACTCG	136	28188	rho GTPase-activating protein 15
atp2b3/pmca3	GCACGAGCAGTGTTGATGTT	TCAACGAACTGACCTGCATC	139	14959	plasma membrane calcium-transporting ATPase 3
atpsyn beta (FW isoform)	TCAAGATGTGCTGCTCTTCG	TGGTGATACGCTCCTGCATA	149	79615	ATP synthase subunit beta, mitochondrial-like
atpsyn beta (SW isoform)	TGCGGGAAGTTGAATCTAGG	ATGTGCCTGCTGATGACTTG	138	1606	ATP synthase subunit beta, mitochondrial-like
casp6	TTTGGCTTTCCAACAAGGTC	CGGAGGCAGATCACTCAGAT	157	24696	caspase-6
ccnb1	ATAATGGGCTGACCAAGCAC	TTGCCAGGTCCTTAACCACT	111	23792	G2/mitotic-specific cyclin-B1
cdc45 variant 1	TTGAACACAAAGAGCAGTTTCG	CACAGATGAAGAAGATGGAGTCA	108	27952	cell division control protein 45 homolog, transcript variant 1
cenpf	GGTGACGGTGCTAGAGAAGG	CTTCCAGTCCAGCAGAGCTT	109	29207	centromere protein F, 350/400kDa (mitosin)
cftr	GGTGCCACAGAAAGTCTTCA	CTTGTCTGGGAACTGCTCAA	139	100895	cystic fibrosis transmembrane conductance regulator
clcc1	GCTCATCCTCAGCAGTTCCT	CTCAACAGCAACATGCCCTA	120	50805	chloride channel CLIC-like protein 1
clcn2/clc-2	GTTATTGCTGGGTGGGTTTG	TGATCCTGTTGGGTTCCATT	149	30	chloride channel protein 2
cldn8	GAGGCTGTAGTGGCTGTTGAG	GGCTTTCTTGTTTGCTGGAG	131	19316	claudin-8
clic4	ACTGGGCGACAGAATGGTAG	GTACCGTGACTTTGCCATCC	137	2581	chloride intracellular channel protein 4
cox3	AAGCCGGTTGCTACAAAGAA	ATCCCTTGCCCTAACCATTC	126	79	cytochrome c oxidase subunit III (mitochondrion)[Hs. homolog]

Gene symbol	5'-Forward primer-3'	5'-Reverse primer-3'	Amplicon size (bp)	Contig	Description
ercc6	CCTCTGTCGGAGAAGCACTC	CTCCATCTCGGAGTCATCGT	122	48759	DNA excision repair protein ERCC-6
foxi2	TGAGTGGAAGAGCTGTGCTG	CGAGGATGACCCAGGTAAAG	137	51168	forkhead box protein I2
gas2	GACATATCGCCAATGATCCA	TGTGCAGCATCCGAATAAAG	114	58776	growth arrest-specific protein 2
gc-1	CCACGCCACTGTAGGTTTCT	ACCCATCCGTCCCTTTCTAT	147	10608	mitochondrial glutamate carrier 1
ghr2	CTGAGGAACAGACCGAGAGG	TCTGCATTCATCACGAGGAG	106	51528	growth hormone receptor II
gpr64	GAAACAGCCACCCAGTTCAT	ACCACAAAGCAGCCAACAG	106	21162	G protein-coupled receptor 64 [Hs. homolog]
gspt2	TCTCTGATGACGTGGAGACG	CAGGTTCTCGGCATTACACA	119	7827	G1 to S phase transition 2
hmx3a	GTTGTTGTTGTGCTCGTCGT	GAGCCAGAGACTCCTCTCCA	113	17416	homeobox protein HMX3-A
map3k15	GCAGGAGAAACATGCCAGAT	CGGTTTCCAGTGCTGATTCT	110	26425	mitogen-activated protein kinase kinase kinase 15
msh2	AATGACGCCTTCACCAGGTA	TGAATGCAGTGTTCCTCTCG	127	1800	DNA mismatch repair protein Msh2
ncc	CACAAAATACCACCCTCGTG	CACAAGCACTACAGGGCAGT	122	11563	Na-Cl cotransporter (NCC)
nd1	AAGGGCCTCCTGCATATTCT	ACTAATCCTTCCCGCCTGAC	140	4520	NADH dehydrogenase subunit 1 (mitochondrion)[Hs. homolog]
nkaa1 (FW isoform)	TCCTGGCTGAACAGAACAAA	AACAGATCCACAGCACAGCTC	104	2955	sodium/potassium-transporting ATPase subunit alpha-1
nkaa1 (SW isoform)	CATGCTGCTGTGGATTGGTGCC	CCGGTGATGATGACGACAGCAGA	129	4958	sodium/potassium-transporting ATPase subunit alpha-1
nkaa3	GGCCGCATTGCTACTCTGACG	ACGCCCAAGAAGACAGCCACT	110	3864	Sodium/potassium-transporting ATPase subunit alpha-3
nkcc1a	GGCCACCAGCCTCCAGAACA	TCAGAGGGACTGTCGGGCTCT	113	12232	Na-K-Cl cotransporter (NKCC1alpha)
rad54l	CCAGGATCTTATGGCTGCAT	GTATCAGGGCTTTGGCGTAG	101	101281	DNA repair and recombination protein RAD54-like
rhgb	AGTGACGGCTCCCACAATAG	TCGGTTTCTTGGCTGGTATC	149	2262	ammonium transporter Rh type B
rock2	GAACGTCTTCAAGCCTCCAC	CAGGAGGAGCAGTTCTTTGG	142	15583	rho-associated protein kinase 2
slc12a6	CGATTGCGCTCATTGATATG	ATCTTGTTCCTGCGGTTGAC	118	39140	solute carrier family 12 member 6
slc14a2/ut2	ACCTGAAACCATTCCCACTG	GCTGTGACAACCCTTGGACT	110	1291	urea transporter 2
slc20a1b	GTATGGTGGCGTGGGTATCT	AGGCCAGTTCGATGCTAAAA	119	2828	sodium-dependent phosphate transporter 1-B

**Gene symbol**	**5’-Forward primer-3’**	**5’-Reverse primer-3’**	**Amplicon size (bp)**	**Contig**	**Description**
slc27a4/fatp4	CTCTGTTCCCTGTGGGACTC	CCAAGGCTGTGATCTTTGGT	128	34423	long-chain fatty acid transport protein 4
slc30a5/znt5	CACGGTCTCCAGGAAGTTGT	AGGACCACGACTCCACTTTG	143	22625	zinc transporter 5
slc30a9/znt9	GTGTAGACCCAAGCCAGGAG	CAGGCCAGAGGACAACATTT	106	17008	zinc transporter 9
slc9a2/nhe2	TTAGGGGCAGCATGTAGAGG	TCAGCATTGGCCTGATAGTG	102	68440	sodium/hydrogen exchanger 2
slc9a3/nhe3	TCTGTACGTGGCTCTCATGG	CAGCAGGAGAAGATCCCAAA	147	6578	sodium/hydrogen exchanger 3
vdac2	GACCAGACCAGAAAGGGTCA	CAGCACACGCTTTGGTATTG	145	6738	voltage-dependent anion-selective channel protein 2
